# Prevention and Treatment of Lower Limb Deep Vein Thrombosis after Radiofrequency Catheter Ablation: Results of a Prospective active controlled Study

**DOI:** 10.1038/srep28439

**Published:** 2016-06-22

**Authors:** Lan Li, Bao-jian Zhang, Bao-ku Zhang, Jun Ma, Xu-zheng Liu, Shu-bin Jiang

**Affiliations:** 1 Coronary care unit, Affiliated hospital of Traditional Chinese Medicine, Xinjiang Medical University, 116 Huanghe Rd, Shayibake District, Urumuqi 830000, China

## Abstract

We conducted a prospective, single-center, active controlled study from July 2013 to January 2015, in Chinese patients with rapid ventricular arrhythmia who had received radiofrequency catheter ablation (RFCA) treatment to determine formation of lower extremity deep vein thrombosis (LDVT) post RFCA procedure, and evaluated the effect of rivaroxaban on LDVT. Patients with asymptomatic pulmonary thromboembolism who had not received any other anticoagulant and had received no more than 36 hours of treatment with unfractionated heparin were included. Post RFCA procedure, patients received either rivaroxaban (10 mg/d for 14 days beginning 2–3 hours post-operation; n = 86) or aspirin (100 mg/d for 3 months beginning 2–3 hours post-operation; n = 90). The primary outcome was a composite of LDVT occurrence, change in diameter of femoral veins, and safety outcomes that were analyzed based on major or minor bleeding events. In addition, blood flow velocity was determined. No complete occlusive thrombus or bleeding events were reported with either of the group. The lower incidence rate of non-occluded thrombus in rivaroxaban (5.8%) compared to the aspirin group (16.7%) indicates rivaroxaban may be administered post-RFCA to prevent and treat femoral venous thrombosis in a secure and effective way with a faster inset of action than standard aspirin therapy.

Over the years, radiofrequency catheter ablation (RFCA) technique has revolutionized the treatment of tachyarrhythmias, and it is considered to be the first-line treatment choice for certain types of arrhythmias. The use of catheter ablation is rationalized by the high success and low complication rates described in the literature^1^,^2^,^3^. Although favorable outcomes have been reported following the use of RFCA, some potential serious complications are still evident. Significant risks include thromboembolic complications, vein vascular endothelial injuries, infection, bleeding, cardiac perforation with or without cardiac tamponade, valvular damage, and risks associated with the procedure or sometimes with the radiation itself^2^,^4^. It has been reported that RFCA may result in the formation of asymptomatic lower limb deep vein thrombosis (LDVT)^5^,^6^. In severe cases, formation of LDVT or femoral venous thrombosis after RFCA may lead to pulmonary thromboembolism or fatal pulmonary embolism (PE)^7^,^8^.

Potential approaches to reduce the risk of thromboembolism include adjunctive administration of platelet inhibitors, intraprocedural intracardiac echocardiography, irrigated radiofrequency ablation, and cryoablation catheter systems^9^; however, anticoagulant therapy remains the mainstay in the treatment of DVT which includes intravenous (IV) infusion of unfractionated heparin (UFH) followed by oral warfarin. Furthermore, subcutaneously administered low-molecular-weight heparin (LMWH) is also considered to be equally effective and safe as IV UFH^10^. Rivaroxaban, an oral, selective inhibitor of factor Xa, is approved by the Food and Drug administration (FDA) for the treatment of DVT and PE, as well as for the reduction in their recurrence risk^11^. The National Institute for Health and Care Excellence (NICE) recommends rivaroxaban as a possible treatment option in adults with DVT and for prevention of DVT recurrence^12^. Moreover, pooled analysis of randomized EINSTEIN-DVT and EINSTEIN-PE trials suggest that rivaroxaban has superior potential in treating veinous thromboembolism which is a collective term for DVT and pulmonary embolism compared to the standard anti-platelet therapy and that it can be used as a single-drug approach for the treatment of DVT and PE^13^.

Although femoral vein remains a crucial emergency venous access route, it is associated with lower extremity DVT after catherization^13^. During RFCA, Multiple venous sheaths are inserted into the femoral vein, which remain in contact with the endothelium for the entire procedural time causing endothelial injury and consequent thrombogenecity leading to fatal DVT and PE^14^. Hence, Patients receiving RFCA therapy should be immediately recommended initial ambulation to prevent the formation of DVT and PE^15^.

Increase in RFCA procedures in recent years has made RFCA-related lower limb thrombosis an important concern. There still exists an unmet need on the occurrence of lower extremity DVT post RFCA procedure and its treatment. Therefore, insights into different treatment aspects are crucial to guide decisions in treatment modalities in susceptible patients during daily practice. In this context, we investigated the effect of rivaroxaban on LDVT post RFCA procedure.

## Results

### Patient baseline demographics and clinical characteristics

**Figure 1:**
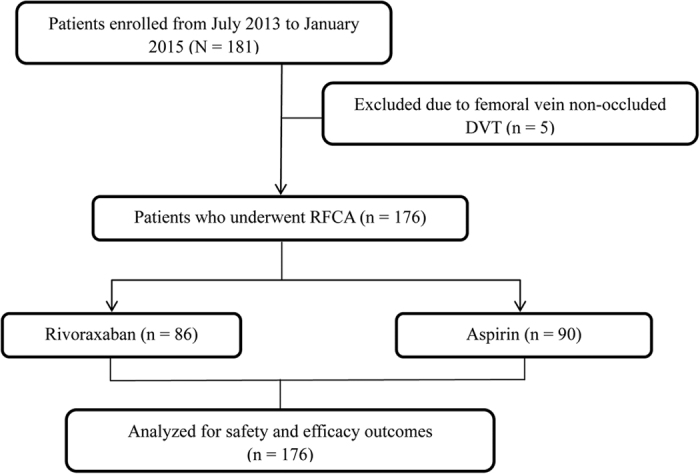
Study flow chart.

Of the selected 181 patients, 5 patients diagnosed with non-occluded DVT in the femoral vein using ultrasound before the surgery were excluded from the study. Data of the remainder 176 patients who underwent the RFCA procedure (male and female patients: n = 83 and 93, respectively; average age: 57.6 ± 14.3 years [13–88 years]) were included for the analysis. In total, 352 bilateral lower limb veins were examined before the RFCA operation (including 176 left and 176 right sides) and 188 femoral veins were examined after the RFCA operation on the punctured sides (including 176 right and 12 left sides), which included 93 in the rivaroxaban and 95 in the aspirin group. No statistical difference was observed in the baseline data of patients in both the groups (*p* > 0.05), as shown in Table 1. The study flow is depicted in Fig. 1.

### Efficacy and safety outcomes

**Figure 2:**
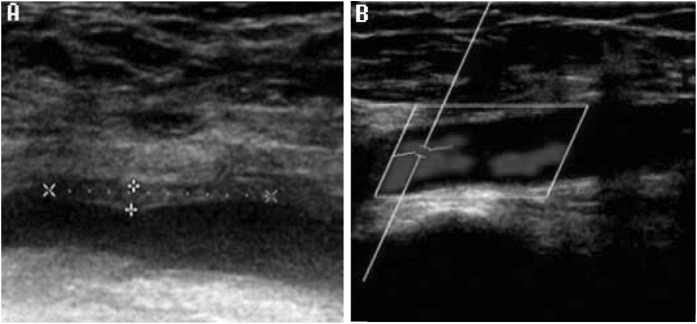
Non-occluded DVT changes of rivaroxaban medical treatment group. (**A**) Formation of femoral vein non-occluded thrombus after RFCA operation. Thrombus is hypoechoic and is generally indistinguishable from flowing blood. Signs indicate the area affected by thrombus (**B**) Longitudinal color Doppler showing disappearance of the femoral vein non-occluded thrombus 14 days after RFCA operation demonstrating normal blood flow.

Of the 188 femoral veins, 176 unpunctured femoral veins were carefully evaluated post RFCA procedure (rivaroxaban, n = 86; aspirin, n = 90). Rivaroxaban significantly decreased the degree of vein stenosis to about 70–80% with an thrombus incidence rate of 5.8% compared to aspirin, which decreased the degree of vein stenosis to about 50–70% with an thrombus incidence rate of 16.7% (*P* = 0.023) (Table 2). Furthermore, no clinical symptoms such as swelling in the lower extremity, pain, and fatigue were observed in both the groups. There were no traces of non-occlusion in the veins following vein ultrasonic re-examination 14 days after the RFCA procedure in patients from both the groups (Fig. 2).

### Changes in the inner diameters of femur veins in patients with thrombosis

There was a decrease in the diameters of femoral portal veins in patients with thrombosis after 48–72 hours of the RFCA procedures compared with those before the procedure (F = 24.966; *p* < 0.001). However, 14 days after the RFCA procedure, thrombosis was not present and the femur diameter of portal veins was not different from those before the operations. After 14 days, the inner diameter of femoral vein was 0.94 ± 0.20 mm in rivaroxaban group and 0.90 ± 0.21 mm in the aspirin group (statistical magnitude [*t*] = 0.37). There was no difference in the femur diameters of portal veins between aspirin and rivaroxaban treatments at 48–72 hours post RFCA procedure (*F* = 2.309; *t* = 0.43; *p* = 0.51), as detailed in Table 3.

### Changes in blood flow rates in the femoral vein

Although the vein flow rates in patients with thrombosis from both the groups were increased 24–72 hours post RFCA procedure compared with those before the procedure, the findings were not statistically significant (*p* > 0.05). After 14 days of the RFCA procedure, blood flow rates in femoral veins were 22.40 ± 2.4 cm/s for the rivaroxaban group and 20.7 ± 4.1 cm/s for the aspirin group (*t* = 0.87; *p* = 0.39). Changes in the femoral vein flow rates of patients in both the groups after the RFCA operation are given in Table 4.

### Safety and other outcomes

None of the 176 patients reported any instance of major or minor bleeding events. There were no untoward effects in the treatment period, including PE such as clinical death, oppression in the chest, expiratory dyspnea, and pectoralgia. An elderly female patient who underwent right femoral vein puncture during the RFCA procedure had frequent ventricular premature beat RFCA in her right ventricular outflow tract and was advised rivaroxaban (10 mg/d) 2–3 hours after the operation for 14 days. There was no abnormality in the right femoral veins 48–72 hours after the operation in any of the patients. However, swelling in the left lower extremity, pain, and activity atony were evident. Lower limb vein digital subtraction angiography on follow-up day 68 revealed left ilium formation of venous thrombosis in a patient, which disappeared after rivaroxaban treatment. None of the remainder patients reported any abnormal discomfort.

## Discussion

The advent of RFCA has significantly changed the therapeutic landscape of supraventricular tachyarrhythmia (SVTA) and has become the first choice of treatment^14^. Most of the definite supraventricular and malignant ventricular arrhythmia may be cured successfully with RFCA procedures^2^,^15^. Although RFCA is a promising technique with minimal invasive surgical technology and high yield-to-complication ratio, there is a likelihood of developing pulmonary thromboembolism and DVT^5^,^8^. The European Multicentre European Radiofrequency Survey (MERFS) results emphasize that the occurrence of pulmonary thromboembolic complications post RFCA procedures cannot be ignored^16^. Reported rates of DVT post endovenous ablation techniques vary widely from 0% to 16%^17^,^18^. In a retrospective analysis, 14 (0.7%) cases of calf-DVT were reported post RFCA procedure^18^. Another retrospective study revealed the risk of post-RFA DVT to be greater in patients with previous DVT^19^. Currently, the rate of RFCA procedures has been increasing and are mostly being conducted through femoral vein^20^,^21^. Literature reveals that RFCA procedures cause venous wounds in the lower limbs and lead to formations of LDVT. Furthermore, results from a randomized trial revealed that femoral vein catheterization is associated with a 25% frequency of LDVT^13^. In this context, physicians are interested in exploring the occurrence of LDVT post RFCA and its treatment options. Therefore, this first, prospective, single-center active controlled study was conducted to determine the rate of LDVT post RFCA procedure and to evaluate the efficacy of rivaroxaban on LDVT.

Miga *et al*. studied 17 patients who underwent RFCA. Anticoagulation therapy was supplied during the procedure (load capacity, 150 μg/kg), and oral aspirin was also administered. Nuclear magnetic resonance angiography 12–70 hours after the operation revealed 4 patients (22%) with altered venous flow (2 complete obstructions and 2 partial obstructions) following catheterization, but no related complications^6^. In another study by Chen *et al*., 52 patients undergoing the RFCA procedure received anticoagulation therapy during left cardiac catheterization (3000–5000 U of heparin, with 1000 U/h). The study revealed 12 veins had non-occluded thrombus, and the incidence rate of formation of femoral venous thrombosis was 17.6%, with no severe clinical symptoms. Follow-up after 7 days revealed no thrombus^5^. Jacobs *et al*. recommended that periprocedural anticoagulation may be considered to reduce the risk of DVT after RFA^19^. Anticoagulation therapy remains the current acceptable treatment option for LDVT^22^. Possible reasons for postoperative thrombosis with propagation to the deep system include undiagnosed hypercoagulable states or local damage to the endothelium from catheter or electrodes^23^. To prevent the occurrence of thrombus, aspirin (oral, 50–150 μg) is usually recommended for a period of l–3 months after RFCA procedure^24^. Rivaroxaban, an oral, highly selective inhibitor of FXa, is characterized by rapid absorption and high biological utilization. Rivaroxaban has no direct effect on platelet aggregation but indirectly inhibits platelet aggregation induced by thrombin and inhibits both free and clot-bound Factor Xa, and prothrombinase activity. FXa inhibition further decreases thrombin generation, consequently increasing clotting times^25^. In addition, age, sex, and weight of the patient do not affect the pharmacokinetics of the drug. Literature supports the administration of rivaroxaban to suppress the occurrence of DVT, with curative effect and function equivalent to low-molecular heparin hypodermal injections^11^,^26^. However, there are no reports regarding the role of the drug in reducing and preventing the occurrence of asymptomatic non-occluded thrombus following RFCA operation.

This study demonstrated that post RFCA procedure, the incidence rate of asymptomatic non-occluded thrombus was 5.8% with rivaroxaban and 15.6% with aspirin. The incidence rate of asymptomatic occlusive thrombus in patients from the aspirin group was similar to that in previously reported literature^5^,^6^. Compared with patients in the aspirin groups, the occurrence of asymptomatic non-occluded thrombus in patients from the rivaroxaban groups is reduced in the punctured vein blood vessels. Furthermore, no major incidences of major or minor bleeding were reported.

Here, we also provided insight on the effect of aspirin in DVT. Although, aspirin is used for DVT prophylaxis and reported to reduce risk of developing blood clots by 30%, few conflicting results has been reported regarding its effectiveness in DVT^27^,^28^,^29^. As per our study, rivaroxaban has produced better results than aspirin; in similar lines Paikin *et al*. stated that physician prefer to use rivaroxaban over aspirin for DVT treatment. Zoy *et al*. compared the efficacy and safety of aspirin, rivaroxaban and low-molecular-weight heparin (LMWH) for DVT post total knee arthroplasty. The incidence of DVT was found to be lesser with rivaroxaban compared with the other two groups [3 (2.94%) vs. 14 (12.50%), P = 0.029; 3 (2.94%) vs. 18 (16.36%), P = 0.017]; however, with increased postoperative blood loss and wound complications. Also, aspirin was recommended as part of a multimodal anticoagulation therapy^30^.

Chen *et al*. demonstrated that the femur diameters of portal veins in the thrombosis portions of non-occluded thrombus were remarkably reduced 1 day after the operation compared with that before the operation. In addition, the femur diameter returned to normal size after 7 days of the operation^5^. In similar lines, this study revealed that the femur diameters of portal veins of thrombosis portions of non-occluded thrombus in patients from both the rivaroxaban and aspirin groups were reduced after 24–72 hours of operation. The degree of coronary artery stenosis was between 20% and 30% with rivaroxaban and 30% and 50% with aspirin. Furthermore, there were no noticeable significant changes in blood flow or any obvious clinical symptoms. The ultrasound re-examination 14 days after the operation demonstrated no thrombus and normal inner diameters of the lumen. Chronically occluded veins have thick walls, are shrunken and often tend to be non-compressible with decreased blood flow rate^31^,^32^. In our study, rivaroxaban had large diameter (48–72 hrs.: 0.86 ± 0.15 mm, 14 days: 0.94 ± 0.20 mm) and better flow rates (48–72 hrs.: 24. 4 ± 7.1 cm/s, 14 days: 22.40 ± 2.4 cm/s ) comparative to aspirin (diameter: 48–72 hrs.: 0.83 ± 0.13 mm, 14 days: 0.90 ± 0.21 mm), (flow rate: 48–72 hrs.: 23.67 ± 3.5 cm/s, 14 days: 20.7 ± 4.1 cm/s) after the RFCA procedure. These observations indicate that rivaroxaban might be more effective in DVT compared with aspirin post RFCA procedure.

A number of studies have demonstrated RFCA to be associated with pre-thrombosis^4^,^33^. Thrombosis may occur inside the heart chamber or in artery and vein blood vessels in the peripheral regions after RFCA^34^. However, a female patient who had no abnormal status in bilateral femoral veins following ultrasonic examination post RFCA procedure developed a venous thrombosis on the non-punctured side after 68 days of follow-up in this study. In this context, the authors suggest that physicians should pay attention not only to the femoral vein blood vessel thrombus on the punctured side but also to thrombotic events on other sites after RFCA.

This study has certain inherent limitations that do not allow for definite conclusions to be drawn. Several elements of the study design may have had an impact on the outcomes. The observations are from a single center with a smaller sample size. Given the concerns mentioned, the conclusions of this study should be treated with caution. Furthermore, well-designed, large-sample, cohort studies and randomized clinical trials are warranted.

## Conclusions

In conclusion, rivaroxaban was found to be effective and safe in post RFCA procedures. It prevents and treats femoral venous thrombosis in a secure and effective way. The study results indicate that rivaroxaban might have a faster onset of action even at low doses compared with aspirin. However, large randomized trials comparing clinical outcomes with rivaroxaban across a wide spectrum of patients are needed to confirm these observations.

## Materials and Methods

### Study design

This was the first, single-center, prospective study to determine the rate of LDVT post RFCA procedure and to evaluate the efficacy of rivaroxaban on LDVT. Between July 2013 to January 2015, 181 patients with rapid ventricular tachyarrhythmia who underwent RFCA treatment in the Coronary Care Unit of Affiliated Hospital of Traditional Chinese Medicine, Xinjiang Medical University, China were included in the study. Of these, 5 patients diagnosed with non-occluded DVT in the femoral vein using ultrasound were excluded from the study. All the patients who had DVT were potentially eligible for the study. Post 2–3 hours of RFCA procedure, patients were assigned to the rivaroxaban group (10 mg/d for 14 days, n = 86) and to the active control aspirin group (100 mg/d for 3 months, n = 90). Patients with asymptomatic LDVT underwent re-examination 10–14 days after the surgery to verify the status of the thrombus. Since the relationship between the dominant LDVT and pulmonary embolism is not clear, there are no corresponding guidelines or expert consensus recommendations for treatment of asymptomatic DVT. Patients with symptomatic LDVT complications received aggressive treatment such as unfractionated heparin, warfarin, low molecular weight heparin.

### Study patients

Patients were selected if they were 18 years or older; had no symptoms of PE; had not received a vitamin-K antagonist or any other anticoagulant; and had received no more than 36 hours of treatment with unfractionated heparin or a low-molecular-weight heparin (3 doses 12 hours apart or 2 doses 24 hours apart). Patients were excluded if they had atrial fibrillation, thrombus or bleeding diseases, coagulation disorders, or hepatic and renal insufficiency; undergone femoral central venous catheter operation for almost 1 month; had documented evidence of recent LDVT; reported cerebral ischemia, intracerebral bleeding, or gastrointestinal bleeding within the past 6 months; or had undergone neurosurgery within the past 4 weeks or other surgery within the past 10 days. Other exclusion criteria were reported evidence of brain metastasis; cytotoxic chemotherapy; life expectancy <6 months; body weight <45 kg; severe heart failure (New York Heart Association class III to IV); uncontrolled severe hypertension (>200/100 mm Hg); and child-bearing potential without effective contraception. Patients were also excluded if they required thrombolytic therapy or treatment with antiplatelet agents, non-steroidal anti-inflammatory drugs with a half-life of >17 hours, or potent CYP3A4 inhibitors, such as ketoconazole. Short-term analgesia with acetylsalicylic acid (500 mg/d) was acceptable before and during the study.

The study protocols conformed to the ethical guidelines of the Declaration of Helsinki and were approved by the institutional review board of Affiliated hospital of Traditional Chinese Medicine, Xinjiang Medical University, China. Written informed consent was obtained from all participants. The study methods were conducted in accordance with approved national and international guidelines.

### Radiofrequency ablation technique

Radiofrequency ablation technique is described elsewhere^13^. Briefly, all surgeries in this study were conducted under general anesthesia (GA) using the Closure system (VNUS medical technologies, San Jose, California). In the conventional RFCA procedure, the sheathing canal in the right femoral vein was inserted by puncturing the subclavian vein, which was fed into the targeted mapping electrode. The sheathing canal in the subclavian vein puncture was introduced into the coronary sinus (CS) electrode by corresponding position and entered into the atrium sinistrum via the atrial septum approach or artery approach. Anticoagulation therapy was administered during the atrium sinistrum procedure and a load capacity of 70–100 U/kg was supplied to the unfractionated heparin (UFH). The dose was increased to 1000 U/kg during the operation; however, UFH was not administered in patients in whom the sheathing canal was introduced via veins. All the procedures were successfully completed.

### Clinical and laboratory indexes

Patients were evaluated for PE symptoms such as clinical death, oppression in the chest, expiratory dyspnea, pectoralgia, blood pressure drop, and right ventricular failure during the study and at scheduled follow-up visits up to 90 days to check for any adverse outcomes. Physicians also evaluated the symptoms of LDVT formation such as swelling in the lower extremity, pain, and activity atony. Before the study, blood, urine, stool routine tests, and also blood chemistry, coagulation, echocardiography samples were collected for all 176 patients enrolled.

### Efficacy and safety outcomes

The primary outcome was a composite of LDVT occurrence (such as swelling in the lower extremity, pains and activity atony), change in diameter of femoral veins, and safety outcomes. All the patients were assessed for the occurrence of LDVT in both the lower limb (by analyzing common femoral vein) and for change in diameter of portal veins before and after 48–72 hours of lower limb RFCA procedure by ultrasound. In addition, blood flow velocity was determined. Color Doppler ultrasound wave (CDU; Germany) with Philips iE33 color Doppler ultrasound diagnostic instrument with linear array 3–8 MHz transducer and Alpha C-arm DSA were used for the assessment. Patients who were found to have venous thrombosis 48–72 hours post RFCA procedure were re-examined after 14 days. Presence of LDVT symptoms during medical treatment was re-examined by CDU to ensure clear diagnosis. To ensure high quality and standardization, all patients were examined in eupneic state by the same professional and well-experienced physician.

Safety was assessed by observing the incidence of major and minor bleeding accidents. Major bleeding events included intracranial hemorrhage, retroperitoneal hemorrhage, intra-spinal bleeding, or reduction in hemoglobin level to ≥5 g/dL. Minor bleeding accidents included bleeding accompanied with decreased hemoglobin level of 3–5 g/dL clinically or bleeding accompanied with decreased hemoglobin level of <3 g/dL clinically.

### Statistical analyses

SPSS software, version 20.0 for windows (SPSS, Inc., Chicago, Illinois), was used to perform statistical analysis. Quantitative variables were expressed by the geometric mean ± standard deviation and were used for comparing two groups. T-inspection was applied for quantitative variables, and the geometric means of two independent samples were compared. Categorical variable rates were assessed by chi-square test. Ordinary chi-square test was applied for inspection when *n* ≥ 40, *T* ≥ 5, whereas calibrated chi-square test was used for inspection when *n* ≥ 40, 1 ≤ *T* < 5. A *p* value of <0.05 was considered to be statistically significant. For unequal variances present between the groups after the t tests, a Levene’s test was used to assess the equality of variances between the means of two or more groups at a significance level α = 0.05, all measurement results are indicated by 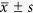
35,36,37.

## Additional Information

**How to cite this article**: Li, L. *et al*. Prevention and Treatment of Lower Limb Deep Vein Thrombosis after Radiofrequency Catheter Ablation: Results of a Prospective active controlled Study. *Sci. Rep.* **6**, 28439; doi: 10.1038/srep28439 (2016).

## Figures and Tables

**Table 1:** Baseline Data of Patients.

Patient characteristics at baseline	Total patients included in the study (n = 176)	Rivaroxaban (n = 86)	Aspirin (n = 90)	*P* value
Gender				
Male, n (%)	83 (47.15)	41 (47.67)	42 (46.66)	0.89
Female, n (%)	93 (52.84)	45 (52.32)	48 (53.33)	0.89
Age (years)	57.6 ± 14.3	55.1 ± 1.38	53.2 ± 1.5	0.37
Patients with atrioventricular nodal re-entrant tachycardia	97 (55.11)	—	—	—
Patients with atrioventricular re-entrant tachycardia	51 (28.97)	—	—	—
Left	39 (76.47)	—	—	—
Right	12 (23.52)	—	—	—
Patients with atrial tachycardia	9 (5.11)	—	—	—
Atrium dextrun AT	4 (44.44)	—	—	—
Atrium sinistrum AT	5 (55.55)	—	—	—
Patients with atrial flutter	5 (2.84)	—	—	—
Patients with ventricular premature beat	10 (5.68)	—	—	—
Left ventricular outflow tract	3 (30)	—	—	—
Right ventricular outflow tract	7 (70)	—	—	—
Patients with ventricular tachycardia	4 (2.27)	—	—	—
Idiopathic right ventricular tachycardia	3 (75)	—	—	—
Idiopathic left ventricular tachycardia	1 (25)	—	—	—
Blood platelet count (10^9^/L)	—	207.7 ± 53.7	211.4 ± 56.7	0.65
PT (s)	—	14.2 ± 5.8	14.3 ± 5.7	.85
PTT (s)	—	32.6 ± 4.3	33.1 ± 3.9	0.42
Smokers, n (%)		32	36	0.70
Glycuresis (number of cases)	—	13	11	0.58
Hypertension (number of cases)	—	31	27	0.39

**Table 2:** Thrombus incident rates of rivaroxaban and aspirin.

Groups	Total Cases	Positive Cases, n (%)	Negative Cases, n (%)	Chi-square	*p*Value
Rivaroxaban	86	5 (5.8)	81 (94.2)	5.14	0.023
Aspirin	90	15 (16.7)	75 (83.3)		
Total		20 (11.4)	168 (88.6)		

**Table 3:** Changes to the inner diameters of femoral veins at different time points.

Group	Before RFCA Operation 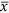 ± *S* (mm)*	48–72 Hours After RFCA Operation 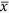 ± *S* (mm)*	14 Days After RFCA Operations 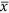 ± *S* (mm)*
Rivaroxaban (5 cases)	1.00 ± 0.256	0.86 ± 0.15	0.94 ± 0.20
Aspirin (15 cases)	0.95 ± 0.267	0.83 ± 0.13	0.90 ± 0.21
*p* value	0.59	0.51	0.58

^*^Results are expressed as (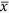 ± *S*) post Levene’s test for unequal variances obtained in t test.

**Table 4:** Changes to the Femoral Vein Flow Rates at Different Time Points.

Group	Cases	Before RFCA Operations 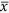 ± *S* (cm/s)*	48–72 Hours after RFCA Operations 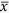 ± *S* (cm/s)*	14 Days After RFCA Operations 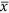 ± *S* (cm/s)*
Rivaroxaban	5	21. 6 ± 2.6	24. 4 ± 7.1	22.40 ± 2.4
Aspirin	15	21.26 ± 5.4	23.67 ± 3.5	20.7 ± 4.1
*p* value		0.895	0.825	0.396

^*^Results are expressed as (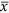 ± *S*) post Levene’s test for unequal variances obtained in t test.

## References

[b1] Cosio F. G. Should ablation be the first line treatment for supraventricular arrhythmias? Heart. 91, 5–6 (2005). 15604319 10.1136/hrt.2004.040121 PMC1768628

[b2] Wellens H. J. Catheter ablation of cardiac arrhythmias: usually cure, but complications may occur. Circulation. 99, 195–7 (1999). 9892581 10.1161/01.cir.99.2.195

[b3] Zipes D. P., DiMarco J. P., Gillette P. C. . Guidelines for clinical intracardiac electrophysiological and catheter ablation procedures. A report of the American College of Cardiology/American Heart Association Task Force on Practice Guidelines (Committee on Clinical Intracardiac Electrophysiologic and Catheter Ablation Procedures), developed in collaboration with the North American Society of Pacing and Electrophysiology. J Am Coll Cardiol. 26, 555–73 (1995). 7608464 10.1016/0735-1097(95)80037-h

[b4] Jesel L., Morel O., Pynn S. . Radiofrequency catheter ablation of atrial flutter induces the release of platelet and leukocyte-derived procoagulant microparticles and a prothrombotic state. Pacing Clin Electrophysiol. 32, 193–200 (2009). 19170908 10.1111/j.1540-8159.2008.02202.x

[b5] Chen J. Y., Chang K. C., Lin Y. C., Chou H. T. & Hung J. S. Safety and outcomes of short-term multiple femoral venous sheath placement in cardiac electrophysiological study and radiofrequency catheter ablation. Jpn Heart J. 45, 257–64 (2004). 15090702 10.1536/jhj.45.257

[b6] Miga D. E., McKellar L. F., Denslow S., Wiles H. B., Case C. L. & Gillette P. C. Incidence of femoral vein occlusion after catheter ablation in children: evaluation with magnetic resonance angiography. Pediatr Cardiol. 18, 204–207 (1997). 9142710 10.1007/s002469900151

[b7] Benito Bartolome F., Sanchez Fernandez-Bernal C. & Prada Martínez F. Pulmonary thromboembolism associated with radiofrequency catheter ablation. Rev Esp Cardiol. 56, 1147–8 (2003). 14622549 10.1016/s0300-8932(03)77028-6

[b8] Haman L., Parizek P., Maly R., Duda J. & Maly J. Analysis of thrombotic complications after catheter ablation. Acta Medica (Hradec Kralove). 49, 47–50 (2006). 16696443

[b9] Zhou L., Keane D., Reed G. & Ruskin J. Thromboembolic complications of cardiac radiofrequency catheter ablation: a review of the reported incidence, pathogenesis and current research directions. J Cardiovasc Electrophysiol. 10, 611–20 (1999). 10355704 10.1111/j.1540-8167.1999.tb00719.x

[b10] Merli G. Anticoagulants in the treatment of deep vein thrombosis. Am J Med. 118 **S8A**, 13S–20S (2005). 16125510 10.1016/j.amjmed.2005.06.006

[b11] Xarelto Prescribing Information. Food and Drug Administration Web site. http://www.accessdata.fda.gov/drugsatfda_docs/label/2013/022406s004lbl.pdfsite (accessed 8th January, 2016).

[b12] Rivaroxaban for the treatment of deep vein thrombosis and prevention of recurrent deep vein thrombosis and pulmonary embolism. National Institute of Health and Care Excellence. https://www.nice.org.uk/guidance/ta261/resources/rivaroxaban-for-the-treatment-of-deep-vein-thrombosis-and-prevention-of-recurrent-deep-vein-thrombosis-and-pulmonary-embolism-82600546953157 (accessed on 8^th^ January, 2016).

[b13] Prins M. H., Lensing A. W., Bauersachs R. . EINSTEIN Investigators. Oral rivaroxaban versus standard therapy for the treatment of symptomatic venous thromboembolism: a pooled analysis of the EINSTEIN-DVT and PE randomized studies. Thromb J. 11, 21 (2013). 24053656 10.1186/1477-9560-11-21 PMC3850944

[b14] Nasrin S., Cader F. A., Salahuddin M., Nazrin T., Iqbal J. & Shafi M. J. Pulmonary embolism as a complication of an electrophysiological study: a case report. Journal of Medical Case Reports. 10, 89 (2016). 27063413 10.1186/s13256-016-0872-0 PMC4827182

[b15] Li Y. C., Lin J., Wu L., Li J., Chen P. & Guang X. Q. Clinical Features of Acute Massive Pulmonary Embolism Complicated by Radiofrequency Ablation: An Observational Study. Medicine (Baltimore). 94, e1711 (2015). 26448025 10.1097/MD.0000000000001711 PMC4616747

[b16] Trottier S. J., Veremakis C., O’Brien J. & Auer A. I. Femoral deep vein thrombosis associated with central venous catheterization: results from a prospective, randomized trial. Crit Care Med. 23, 52–9 (2005). 8001386 10.1097/00003246-199501000-00011

[b17] Fuster V. . 2011 ACCF/AHA/HRS focused updates incorporated into the ACC/AHA/ESC 2006 Guidelines for the management of patients with atrial fibrillation: a report of the American College of Cardiology Foundation/American Heart Association Task Force on Practice Guidelines developed in partnership with the European Society of Cardiology and in collaboration with the European Heart Rhythm Association and the Heart Rhythm Society. J Am Coll Cardiol. 57, e101–98 (2011). 21392637 10.1016/j.jacc.2010.09.013

[b18] Cardiovascular Disease Branch of Chinese Medical Association tCPaEBoCSoBE, Editorial Board of Chinese Journal of Cardiac Pacing and Electrophysiology, Editorial Board of Chinese Journal of Cardiology. Guidance on the treatment of super-aventricular tachyarrhythmia. Guidance on the treatment of super-aventricular tachyarrhythmia. Chinese Journal of Cardiac Pacing and Electrophysiology. 19, 3–15 (2005).

[b19] Hindricks G. The Multicentre European Radiofrequency Survey (MERFS): complications of radiofrequency catheter ablation of arrhythmias. The Multicentre European Radiofrequency Survey (MERFS) investigators of the Working Group on Arrhythmias of the European Society of Cardiology. Eur Heart J. 14, 1644–1653 (1993). 8131762 10.1093/eurheartj/14.12.1644

[b20] Mozes G., Kalra M., Carmo M., Swenson L. & Gloviczki P. Extension of saphenous thrombus into the femoral vein: a potential complication of new endovenous ablation techniques. J Vasc Surg. 41, 130–135 (2005). 15696055 10.1016/j.jvs.2004.10.045

[b21] Marsh P., Price B. A., Holdstock J., Harrison C. & Whiteley M. S. Deep vein thrombosis (DVT) after venous thermoablation techniques: rates of endovenous heat-induced thrombosis (EHIT) and classical DVT after radiofrequency and endovenous laser ablation in a single centre. Eur J Vasc Endovasc Surg. 40, 521–527 (2010). 20655773 10.1016/j.ejvs.2010.05.011

[b22] Jacobs C. E., Pinzon M. M., Orozco J., Hunt P. J., Rivera A. & McCarthy W. J. Deep venous thrombosis after saphenous endovenous radiofrequency ablation: is it predictable? Ann Vasc Surg. 28, 679–685 (2014). 24211409 10.1016/j.avsg.2013.08.012

[b23] Alizadeh A., Yazdi A. H., Kafi M., Rad M. A., Moradi M. & Emkanjoo Z. Predictors of local venous complications resulting from electrophysiological procedures. Cardiol J. 19, 15–19 (2012). 22298163 10.5603/cj.2012.0004

[b24] Tompkins C. Venous thrombosis following femoral venous access for electrophysiology studies: an on-going challenge. Cardiol J. 19, 1–3 (2012). 22298160 10.5603/cj.2012.0001

[b25] Masuda E. M., Kistner R. L., Musikasinthorn C., Liquido F., Geling O. & He Q. The controversy of managing calf vein thrombosis. J Vasc Surg. 55, 550–561 (2012). 22032881 10.1016/j.jvs.2011.05.092

[b26] Hingorani A. P. . Deep venous thrombosis after radiofrequency ablation of greater saphenous vein: a word of caution. J Vasc Surg. 40, 500–504 (2004). 15337880 10.1016/j.jvs.2004.04.032

[b27] Cardiac Pacing and Electrophysiology Branch of Chinese Society of Biomedical Engineering, Cardiac Pacing and Electrophysiology Branch of Chinese Medical Association, Editorial Office of Chinese Journal of Cardiac Pacing and Electrophysiology. Guidance on the Radio frequency catheter ablation of rapid ventricular arrhythmia (revised edition). Chinese Journal of Cardian Pacing and *Electrophysiology*. **16**, 85-91 (2002).

[b28] Samama M. M. The mechanism of action of rivaroxaban – an oral, direct Factor Xa inhibitor – compared with other anticoagulants. Thrombosis research. 127, 497–504 (2011). 20888031 10.1016/j.thromres.2010.09.008

[b29] Dillier R. . Safety of continuous periprocedural rivaroxaban for patients undergoing left atrial catheter ablation procedures. Circ Arrhythm Electrophysiol. 7, 576–582 (2014). 24970293 10.1161/CIRCEP.114.001586

[b30] Paikin J. S. & Eikelboom J. W. Aspirin. Circulation. 125, e439–e442 (2012). 22412097 10.1161/CIRCULATIONAHA.111.046243

[b31] Pulmonary Embolism Prevention (PEP) trial Collaborative Group. Prevention of pulmonary embolism and deep vein thrombosis with low dose aspirin: Pulmonary Embolism Prevention (PEP) trial. *Lancet*. **355**, 1295–1302 (2000). 10776741

[b32] Goldhaber S. Z. & Fanikos J. Prevention of Deep Vein Thrombosis and Pulmonary Embolism. Circulation. 110, e445–e447 (2004). 15492324 10.1161/01.CIR.0000145141.70264.C5

[b33] Zou Y., Tian S., Wang Y. . Administering aspirin, rivaroxaban and low-molecular-weight heparin to prevent deep venous thrombosis after total knee arthroplasty. Blood Coagul Fibrinolysis. 25, 660–4 (2014). 24695091 10.1097/MBC.0000000000000121

[b34] Meissner M. H., Moneta G. & Burnand K. . The hemodynamics and diagnosis of venous disease. J Vasc Surg. 46, 4S–24S (2007). 18068561 10.1016/j.jvs.2007.09.043

[b35] Bandyopadhyay G., Roy S. B., Haldar S. . Deep vein thrombosis. J Indian Med Assoc. 108, 866–7 (2010). 21661467

[b36] Dorbala S., Cohen A. J., Hutchinson L. A., Menchavez-Tan E. & Steinberg J. S. Does radiofrequency ablation induce a prethrombotic state? Analysis of coagulation system activation and comparison to electrophysiologic study. J Cardiovasc Electrophysiol. 9, 1152–1160 (1998). 9835258 10.1111/j.1540-8167.1998.tb00086.x

[b37] Thakur R. K., Klein G. J., Yee R. & Zardini M. Embolic complications after radiofrequency catheter ablation. Am J Cardiol. 74, 278–279 (1994). 8037136 10.1016/0002-9149(94)90373-5

